# Socioeconomic and urban-rural differentials in exposure to air pollution and mortality burden in England

**DOI:** 10.1186/s12940-017-0314-5

**Published:** 2017-10-06

**Authors:** Ai Milojevic, Claire L. Niedzwiedz, Jamie Pearce, James Milner, Ian A. MacKenzie, Ruth M. Doherty, Paul Wilkinson

**Affiliations:** 1 Department of Social and Environmental Health Research, London School of Hygiene & Tropical Medicine, 15-17 Tavistock Place, London, WC1H 9SH UK; 2 Centre for Research on Environment Society and Health, School of GeoSciences, University of Edinburgh, Drummond Street, Edinburgh, EH8 9XP UK; 3 School of GeoSciences, University of Edinburgh, James Hutton Road, Edinburgh, EH9 3FE UK

**Keywords:** Socioeconomic inequalities, Air pollution, Health burdens, Fine particles, Life years lost, Mortality, England

## Abstract

**Background:**

Socioeconomically disadvantaged populations often have higher exposures to particulate air pollution, which can be expected to contribute to differentials in life expectancy. We examined socioeconomic differentials in exposure and air pollution-related mortality relating to larger scale (5 km resolution) variations in background concentrations of selected pollutants across England.

**Methods:**

Ozone and particulate matter (sub-divided into PM_10_, PM_2.5_, PM_2.5–10_, primary, nitrate and sulphate PM_2.5_) were simulated at 5 km horizontal resolution using an atmospheric chemistry transport model (EMEP4UK). Annual mean concentrations of these pollutants were assigned to all 1,202,578 residential postcodes in England, which were classified by urban-rural status and socioeconomic deprivation based on the income and employment domains of the 2010 English Index of Multiple Deprivation for the Lower-level Super Output Area of residence. We used life table methods to estimate PM_2.5_-attributable life years (LYs) lost in both relative and absolute terms.

**Results:**

Concentrations of the most particulate fractions, but not of nitrate PM_2.5_ or ozone, were modestly higher in areas of greater socioeconomic deprivation. Relationships between pollution level and socioeconomic deprivation were non-linear and varied by urban-rural status. The pattern of PM_2.5_ concentrations made only a small contribution to the steep socioeconomic gradient in LYs lost due to PM_2.5_ per 10^3^ population, which primarily was driven by the steep socioeconomic gradient in underlying mortality rates. In rural areas, the absolute burden of air pollution-related LYs lost was lowest in the *most* deprived deciles.

**Conclusions:**

Air pollution shows modest socioeconomic patterning at 5 km resolution in England, but absolute attributable mortality burdens are strongly related to area-level deprivation because of underlying mortality rates. Measures that cause a general reduction in background concentrations of air pollution may modestly help narrow socioeconomic differences in health.

**Electronic supplementary material:**

The online version of this article (10.1186/s12940-017-0314-5) contains supplementary material, which is available to authorized users.

## Background

There is consistent evidence of the long-term effects of air pollution on mortality [1–6]. The Committee on the Medical Effects of Air Pollutants (COMEAP) estimated that, at 2008 levels, fine particle pollution produced by human activity (anthropogenic PM_2.5,_ particles with diameter < 2.5 μm) had an annual effect on UK mortality equivalent to around 340,000 life-years (LYs) lost in those aged over 30 years, which is equivalent to an average loss of life expectancy from birth of approximately six months, or around 9 min (0.65%) from each day of life [7]. Although the COMEAP report did not quantify, the degree to which such mortality burdens may vary by socioeconomic status. According to National Statistics, the gap in life expectancy between the least and most deprived populations in England and Wales is around 6.7 years for men and 5.3 years for women based on the Slope Index of Inequality [8], differences which may in part reflect the influence of environmental factors amongst others.

Generally, socioeconomic inequalities in health are thought to reflect two major mechanisms, differential distribution of exposure and differential susceptibility, acting independently or together [9–12]. Susceptibility may be divided into susceptibility that is captured by the (multiplying effect of) underlying health/mortality rates, and susceptibility that arises from an intrinsic vulnerability of the individual (specifically, sensitivity) that means a given level of air pollution exposure has a greater relative impact in more deprived than in less deprived populations [12, 13]. Differential exposure and perhaps differential sensitivity to air pollution might partially contribute to underlying mortality rates, but we believe it is marginal.

Assessing how environmental exposure may partly explain general health inequalities has been a major subject of public health research. There is significant body of evidence from countries such as the United States, Canada, Sweden, New Zealand and the UK demonstrating that low income individuals and residents of more socially disadvantaged areas tend to be exposed to higher levels of air pollutants including nitrogen dioxide (NO_2_) and PM_10_ (particles <10 μm) [14–19]. A recent review of the global evidence of the unequal exposure of environmental hazards (including air pollution) on disadvantaged and vulnerable populations showed that the majority of studies in North America demonstrate that residents of low socioeconomic status communities experience higher concentrations of air pollution, but the findings in Europe are more equivocal [9, 20]. Such differentials in exposure are likely to contribute modestly to the strong social gradients in health observed in many settings.

Differentials in susceptibility may reflect the influence of many factors including material deprivation (access to health care or fresh foods), psychosocial stress, underlying health conditions and risky behaviours. In the air pollution literature, susceptibility (specifically, sensitivity) has been commonly evaluated in the form of effect modification but evidence remains mixed. A recent US cohort study (the Women’s Health Initiative Observational Study) found socioeconomic status did not confound the positive association between PM_2.5_ and CVD, but modified the effect (i.e. higher CVD risk due to PM_2.5_ exposure for women living in more disadvantaged neighbourhoods) [21]. Other supportive evidence of the synergistic adverse effects of air pollution and socioeconomic factors includes reports from the US, Canada, Italy, Switzerland, Hong Kong and Latin America [22–28]. On the other hand, a cross-sectional analysis of a large population-based US cohort of adults without a history of CVD (the Multi-Ethnic Study of Atherosclerosis) found little evidence that social disadvantage confers increased sensitivity to the hypertensive effects of PM_2.5_ [29], which is consistent with several other previous studies [30–33]. These studies commonly reported multiplicative scale interactions (i.e. difference in relative risks across population subgroups) to demonstrate differential susceptibility. However, such studies have not in general reported on the impact of differences in underlying rates of the morbidity/mortality [12]. The impact of such differentials is clear when results are reported on an absolute (difference) scale - i.e. in terms of numbers of deaths or hospitalizations attributable to air pollution exposure [12].

In this paper, we aim to combine two dimensional socioeconomic differentials (in air pollution exposure and susceptibility), if any, by the use of health impact assessment in order to demonstrate the extent to which socioeconomic differentials in exposures contribute to the socioeconomic gradient in those health impacts. Specifically, we (1) characterize the association between annual average exposure to air pollution, socioeconomic deprivation and urban-rural status; and (2) estimate associated mortality burdens based on observed socioeconomic and urban-rural differentials in exposure and underlying mortality.

## Methods

### Modelled exposure to air pollution

Annual mean concentrations of ozone (O_3_) and particulate matter (PM) were derived at 5 km horizontal resolution in England (5683 grids) from values computed hourly by the European Monitoring and Evaluation Programme (EMEP) for the UK atmospheric chemistry transport model for 2010. Full details of EMEP4UK model are described elsewhere [34, 35]. Briefly, it is a UK-focussed, nested version of the EMEP MSC-W (version 4.3) model for Europe [36] with increased horizontal resolution (5 km) over an inner British Isles domain. It is driven by sub-hourly meteorology from the Weather Research and Forecast (WRF) model version 3.1.1 (https://www.mmm.ucar.edu/weather-research-and-forecasting-model). WRF is continuously constrained (every 6 h) to observed meteorological parameters ensuring that it represents close-to-real weather conditions throughout the simulations. Anthropogenic emissions are derived from the UK National Atmospheric Emission Inventory (NAEI). The main advantages of a high resolution atmospheric chemistry transport model (CTM) for studying air pollution epidemiological studies include fine temporal and spatial resolution covering the whole UK, and provision of data on individual particle chemical components. Ground-level modelled concentrations of components were calculated hourly at 3 m above the surface vegetation or other canopy. Anthropogenic and natural PM in EMEP4UK is modelled in two size categories PM_2.5_ and PM_2.5–10_ (particles with diameter in the range 2.5 to 10 μm), which together make up PM_10_. Total PM_2.5_ consists of ammonium (NH_4_^+^), sulphate (SO_4_^2−^), nitrate (NO_3_^−^), elemental carbon (EC), organic matter (OM), non-carbonaceous primary, sea salt (SS) and mineral dust. PM_2.5–10_ contains the same constituent species (in different proportions) as PM_2.5_ without NH_4_^+^ and SO_4_^2−^ which exist in the model as fine particles only. These components include both primary (directly emitted) particles (e.g, EC) and secondary particles formed within the atmosphere from gaseous precursors (e.g, SO_4_^2−^). Of these components we examined the fine fractions of sulphate, nitrate and primary anthropogenic (OM and EC from fossil fuel combustion and remaining non-carbonaceous primary) together with total PM_2.5_, PM_2.5–10_ and PM_10_. Exposure was characterized by the annual mean of daily means (annual mean of daily maximums of 8-h running means for O_3_). Spatial distribution of these exposure measures are presented as maps in Additional file 1.

### Measurement of socioeconomic deprivation

The socioeconomic deprivation index was modified from the 2010 English Index of Multiple Deprivation (IMD) [37] which is a weighted composite of small area data combining seven domains: Income; Employment; Health and Disability; Education, Skills and Training; Barriers to Housing and Services; Crime; and Living Environment.

Specifically, we excluded the Health and Disability domain and the Living Environment domain which partially included variables to be incorporated in the main analytical model (small-area statistics of mortality and ambient concentration of PM and other air pollutants, respectively), and reconstructed the deprivation index from the key domains (i.e. the Income domain and the Employment domain) only, keeping the original proportion of weights (equal weight for each domain) at the Lower-level Super Output Area (LSOA), following the approaches used in previous studies [38–40]. This is to avoid the duplication in explanatory and dependent variables in the analysis, though high correlation between the original and modified deprivation index (Pearson’s *r* = 0.95) suggests little effect on the main results.

LSOA is a small area unit designed to be socially homogeneous and has a relatively even population size with 1500 residents on average (precisely 1000 to 3000 population), but varies in its areal size (mean 1.0 ± SD 2.4 km^2^ in urban area and 19 ± 27.8 km^2^ in rural area). All LSOAs in England (*n* = 32,179) were classified into decile groups according to the reconstructed deprivation index to keep equal population numbers across the groups. As such, the decile group 1 represents the least deprived 10% of residents in England and group 10 indicates the most deprived 10% (see a map of deprivation decile groups in Additional file 1).

### Data linkage

Residential unit postcodes (on average 15 addresses per unit, *n* = 1,202,578 in England) were selected from Codepoint Postcode data (Office for National Statistics, ONS) and linked to annual level of air pollution (stated above), residential population (2011 Census, Headcount and Household Estimates for postcodes in England and Wales) [41], all death events sourced from the deaths register (post-coded, ONS) in 2006 through residential address of the deceased to calculate baseline mortality (described below), and other areal markers such as socioeconomic deprivation groups (stated above) and LSOA Rural-Urban Classification (ONS, based on the definition of urban area as physical settlements with a population of 10,000 or more) [42]. Data linkage of unit postcodes with the 5 km EMEP4UK grids and LSOAs were processed by the overlay (intersect) function of ArcGIS (version 10.3) at the closest delivery point to the calculated mean position of all the delivery points in the area unit.

### Analysis

Our analyses consisted of two steps: 1) examination of the socioeconomic gradient in air pollution exposure and 2) quantification of the associated mortality impact. First, population-weighted averages of air pollution levels were estimated by a regression model of all residential postcodes using categorical variables of deprivation groups, adjusting for region to control for previously reported north-south differences in association between deprivation and mortality. This is to make sure our estimated socioeconomic differentials (if any) in exposure to air pollution is independent from regional effects. Confidence intervals (CIs) for estimated population-weighted averages of air pollution levels were adjusted for possible noise from clustering by district using Huber-White variance [43].

Next, in order to quantify the mortality impact associated with derived exposure levels of pollution, a standard life table calculation method [44] was applied to estimate LYs lost. Here, only the effect of PM_2.5_ was considered using a concentration-response relationship from the results of the American Cancer Society (ACS) cohort studies in the US (relative risk of all-cause mortality of 1.06 per 10 μg/m^3^ increase in PM_2.5_) [4] following the health impact assessment method conducted by COMEAP [7], We assumed no effect of PM_2.5_ exposure for those aged under 30 years to be consistent with the reported concentration-response relationship. Baseline mortality rates were calculated specifically for gender, five year age groups, decile of deprivation and urban/rural groups for all-cause deaths registered in the official statistics (ONS) in 2006, England and mid-year population estimates by LSOA in the same year [45]. 2006 was the latest available year of appropriate death registry and population data for this study. Given that baseline mortality rates in England will have changed very little over the period 2006–2010, the 2006 data provide a good approximation for 2010. For the purpose of comparing baseline mortality rate across deprivation groups, age-standardized death rates (ASDR) were calculated by direct standardization method. Absolute and relative measures of associated mortality burdens were presented in terms of total LYs lost and LYs lost per 1000 population, respectively, across deprivation groups and by urban-rural status. PM_2.5_ exposure-related life expectancy lost at birth was also presented for the purpose of comparison with the reported overall life expectancy [8]. Regression analyses were performed with Stata version 14 and life table calculations using a set of linked spread sheets in Microsoft Excel 2013.

## Results

### Air pollution exposure patterns

Annual mean concentrations of air pollution (simulated for 2010) were assigned to the 1.2 million residential postcodes in England, of which a quarter were classified as rural. Generally, concentrations of total PM_2.5_ and PM_10_, sulphate and primary PM_2.5_ were higher in urban than in rural areas based on both simple arithmetic mean and population-weighted mean pollution levels; the reverse was true for O_3_, nitrate PM_2.5_ and, for more deprived areas, PM_2.5–10_ (Table 1).

**Table 1:** Summary statistics

	All areas	Urban areas	Rural areas
Residential postcodes: n (%)	1,202,578	886,683	315,895
	(100%)	(74%)	(26%)
LSOA: n (%)	32,179	26,632	5557
	(100%)	(83%)	(17%)
Population in mid-2006: n (%)	52,122,136	43,140,763	8,981,373
	(100%)	(83%)	(17%)
2010 annual mean concentration (arithmetic mean), μg/m^3^: mean (sd)			
Total PM_2.5_	9.21 (0.71)	9.32 (0.66)	8.89 (0.75)
Nitrate PM_2.5_	1.91 (0.25)	1.90 (0.23)	1.95 (0.31)
Sulphate PM_2.5_	1.49 (0.17)	1.51 (0.17)	1.42 (0.17)
Primary PM_2.5_	1.44 (0.39)	1.55 (0.37)	1.12 (0.27)
PM_2.5–10_	7.98 (0.63)	7.97 (0.53)	8.01 (0.84)
PM_10_	17.19 (0.92)	17.29 (0.86)	16.89 (1.01)
O_3_	68.46 (3.54)	67.66 (3.47)	70.69 (2.68)
2010 annual mean concentration (population-weighted mean), μg/m^3^			
Total PM_2.5_	9.30	9.37	8.95
Nitrate PM_2.5_	1.91	1.90	1.97
Sulphate PM_2.5_	1.50	1.51	1.44
Primary PM_2.5_	1.51	1.58	1.15
PM_2.5–10_	7.96	7.96	7.97
PM_10_	17.26	17.33	16.92
O_3_	68.04	67.53	70.46

**Fig. 1:**
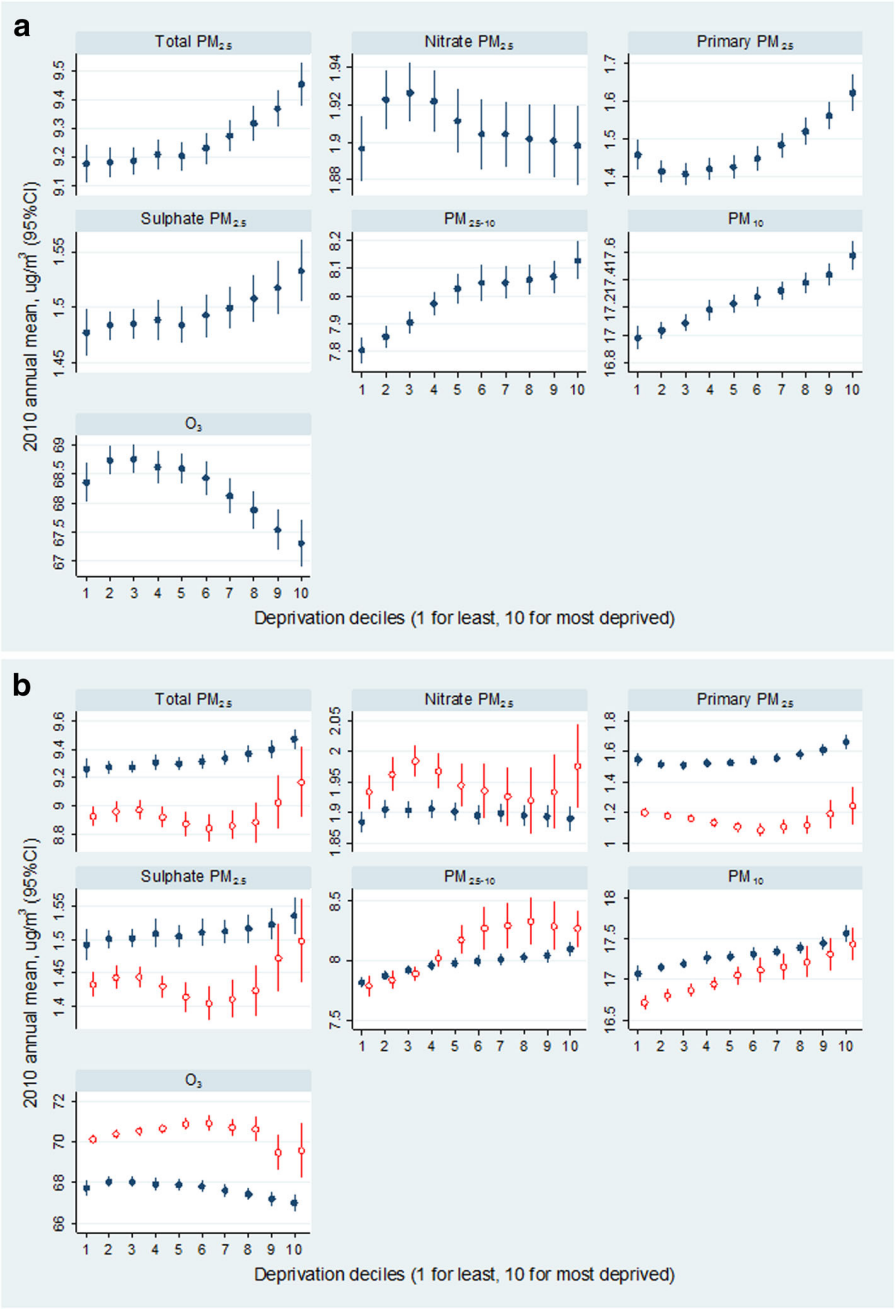
Patterns of concentrations of PM fractions and O_3_ in England: **a** by decile of socioeconomic deprivation and (**b**) by socioeconomic and urban-rural status. Annual mean concentrations were estimated by regression models allowing for district-level clustering with adjustment for region

Variations of mean pollutant concentrations by decile of IMD were apparent for all pollutants, including individual PM_2.5_ components (Fig. 1a). Traffic-related air pollution (primary and total PM_2.5_) showed higher concentrations in the areas of greater socioeconomic deprivation: the ratios of the most versus the least deprived decile group were 1.11 and 1.03. For example, estimated total PM_2.5_ level for the most deprived quintile group (9.45 μg/m^3^) was 3% higher than that for the least deprived group (9.18 μg/m^3^). There was a similar pattern of higher concentrations with greater deprivation for PM_10_, PM_2.5–10_ and sulphate. In contrast, O_3_ showed a pattern of generally decreasing concentrations as socioeconomic deprivation increased). Except for PM_10_, the socioeconomic gradients in pollutants did not appear to be simple linear relationships, there usually being differences in the patterns between the five least and five most deprived deciles.

The socioeconomic patterns of air pollution concentrations varied by urban-rural status (Fig. 1b), with variations by decile of IMD being generally slightly larger in rural areas. For example, the concentration of total PM_2.5_ was 0.32 (95%CI 0.09, 0.55) μg/m^3^ greater in decile 10 compared with decile 6 in rural areas, while the equivalent difference in urban areas was 0.16 (0.11, 0.21) μg/m^3^. For O_3_, the difference in concentration for decile 10 compared with decile 6 was −1.34 (95%CI -2.62, −0.06) μg/m^3^ in rural areas and −0.80 (−1.08, −0.51) μg/m^3^ in urban areas. Socioeconomic patterns in traffic related air pollution (namely total PM_2.5_) were slightly U-shaped in rural areas (Fig. 1b), with a fall in concentration among the five least deprived deciles followed by a small increase among the five most deprived groups: −0.05 μg/m^3^ and +0.32 μg/m^3^ per change in deprivation decile group 1 to 5 and 6 to 10 for total PM_2.5_ in rural areas. The different size fractions of PM showed distinct concentration patterns in rural areas – e.g. a relatively steep socioeconomic gradient across the five most deprived deciles of PM_2.5_ (a difference of 0.32 μg/m^3^ between decile 6 and 10), whereas for PM_2.5–10_, the gradient was steepest in the five least deprived deciles (a change of 0.39 μg/m^3^ for decile 5 compared with decile 1). Interestingly, urban-rural differentials in concentrations of PM_2.5–10_ (higher PM_2.5–10_ in rural area) were obvious only in the more deprived decile-groups and the less deprived deciles in rural areas exhibit similar PM_2.5–10_ levels as urban areas.

### Associated mortality burden

**Fig. 2:**
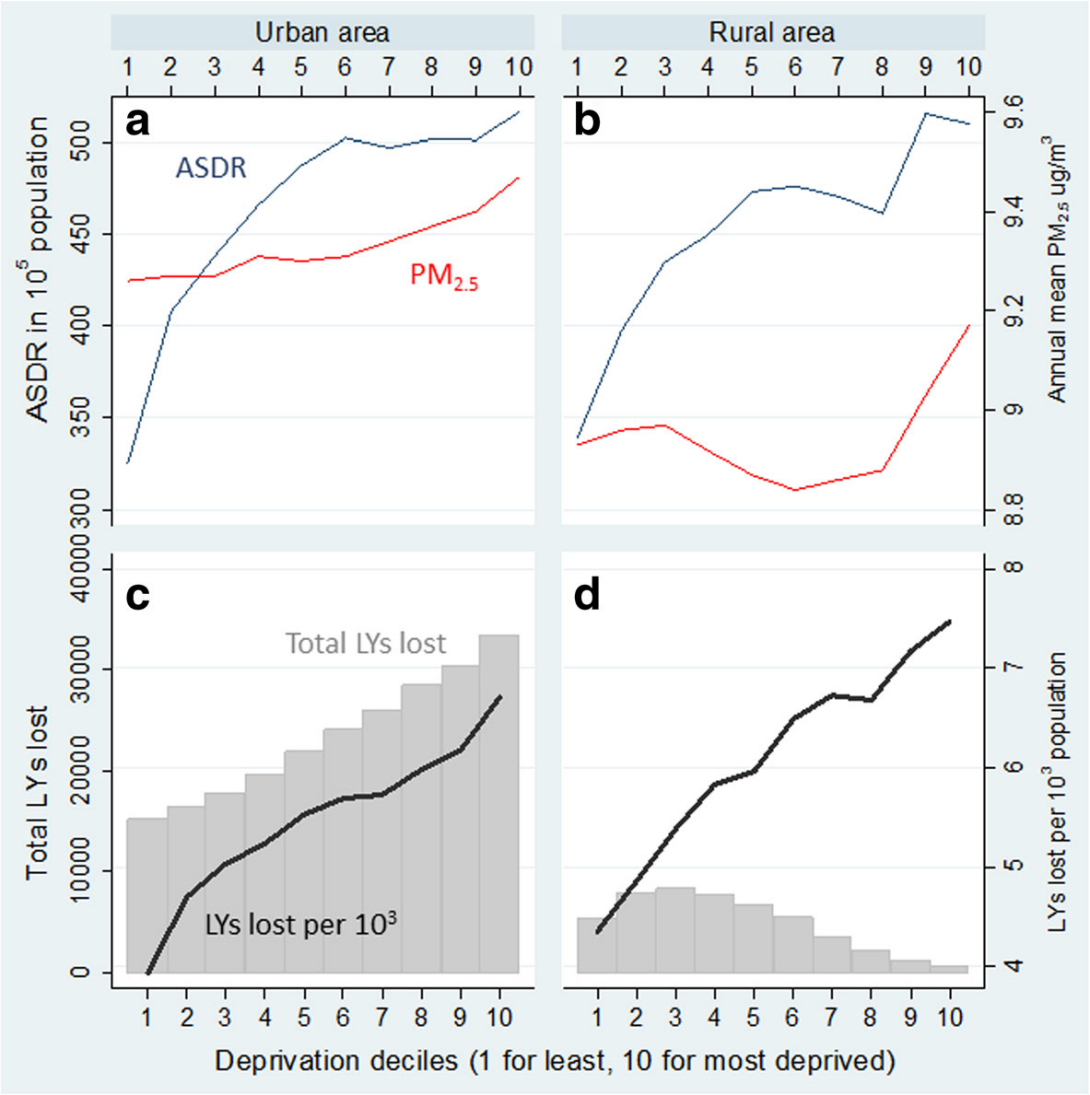
Observed annual average PM_2.5_ concentrations (red lines) and age-standardized death rate (ASDR in blue lines) by decile of socioeconomic deprivation for urban (**a**) and rural (**b**) areas. Corresponding patterns of life years (LYs) lost associated with PM_2.5_ (total (grey bars) and per 10^3^ population (thick black lines)) (panels (**c**) and (**d**))

The total LYs lost attributable to long-term exposure to PM_2.5_ in England, estimated for 2010, were 283,084 years, of which 82% (233,257 years) were in urban areas and 18% (49,804 years) in rural areas. PM_2.5_-related LYs lost by decile of IMD are presented in Fig. 2, along with baseline mortality (ASDR) and annual mean total PM_2.5_ concentrations (see Additional file 2 for corresponding table).

The ratios of the most deprived against the least deprived decile in ASDR were 1.59 and 1.50 in urban and rural areas, respectively; by comparison, the corresponding ratios for total PM_2.5_ concentration were much smaller at just 1.02 and 1.03 (Fig. 2a and b).

There were steep increases with deprivation in PM_2.5_-related LYs lost per 10^3^ population in both urban and rural areas, but the total LYs lost showed a strong, broadly linear, increase with deprivation only in urban areas (Fig. 2c and d). In urban areas, the total LYs lost in the most deprived group were 18,200 greater than the least deprived, corresponding to a ratio of burdens in the most to the least deprived group of 2.2 (see Additional file 2). In rural areas, however, there was a pattern of modest rise then fall in total attributable LYs lost across deciles and total LYs lost in the most deprived group were 4700 less than the least deprived group (corresponding ratio of 0.13). Only 1–2% of those in the most deprived 20% of population in England live in rural area (Additional file 2).

These contrasts suggest there were substantial gradients in attributable burdens per 10^3^ population despite only modest differences in air pollution (PM_2.5_) concentrations across deciles of deprivation because air-pollution related relative risks are applied to substantial differences in the underlying mortality rates.

The differentials in PM_2.5_ exposure-related life expectancy lost between least and most deprived areas was 0.13 years (47 days) for men and 0.12 years (46 days) for women in England overall (see Additional file 3). This is in contrast to the reported overall life expectancy lost due to socioeconomic differentials, 6.7 years and 5.3 years for men and for women, respectively [8].

## Discussion

### Summary of findings

This study has quantified socioeconomic differentials in broader scale geographical variations in the annual average background concentration of particulate matter and ozone in England, and in the PM_2.5_-associated impacts on mortality. Concentrations of the most particulate fractions, but not of nitrate PM_2.5_ or ozone, were modestly higher in areas of greater socioeconomic deprivation though pollution relationships were non-linear and varied by urban-rural status. However, such pollution differentials made only a small contribution to socioeconomic gradients in PM_2.5_-attributable LYs lost per 10^3^ population in both urban and rural areas. Our analyses suggest that such gradients *are* substantial but mainly because of the gradient in underlying mortality rates across deciles of deprivation (which therefore generate gradients in morality burdens even with a similar *relative* risk for PM_2.5_), rather than because of variations in pollution concentrations themselves. The small mortality burden of PM_2.5_ exposure in the most deprived decile in rural areas is due to the small population in such areas. The magnitude of the socioeconomic differentials in PM_2.5_-related life expectancy lost at birth is fairly modest by comparison with the differentials in overall life expectancy between least and most deprived areas.

### Comparison with other studies

Socioeconomic variations in exposure to ambient particulate air pollution in the UK have been described in a number of settings [46–50], including the longitudinal studies examined the socioeconomic distribution of the air quality improvement over the last decade [51, 52].

A comprehensive study on spatial inequality in England found weak association between population-weighted PM_10_ concentration and IMD in England 2004, which differs to some extent between urban and rural area and at different geographical scales (stronger associations with increasing level of spatial aggregation) [50]. Another UK wide study reported U-shaped association between income deprivation and population-weighted PM_10_ or NO_2_ (averaged 1999–2003), but at a lower spatial resolution (Census Area Statistics wards with mean population size 5518) [47]. Studies in four conurbations (London, Birmingham, Cardiff and Belfast) in the UK reported higher NO_2_ and PM_10_ concentrations in areas exhibiting higher levels of deprivation in all cities except Cardiff [48].

The results of our study provide additional evidence of such variations in relation to a range of particle sub-fractions and, more importantly, the contribution of air pollution to socioeconomic differentials in mortality based on PM_2.5_. The results are important in demonstrating that though air pollution differentials exist with respect to socioeconomic status, they are generally modest, at least at the geographical resolution used in this study (5 km horizontal grid). Consequently, the impact of air pollution on mortality across deciles of socioeconomic status shows little variation in *relative* terms. Yet there is a substantial gradient in attributable LYs lost per 10^3^ population because of the magnitude of the socioeconomic gradient in underlying age-specific mortality rates. Another study which examined the prognosis of acute coronary syndrome (ACS) in England and Wales also found a marginal contribution of exposure to air pollution to socioeconomic inequalities in survival among patients with a previous ACS event [53].

### Advantages and limitations of this study

One of the main advantages of this study is the use of nation-wide data of air pollution and socioeconomic deprivation markers covering the whole of England, which enabled us to investigate not only urban areas but also rural areas of which air pollution levels are often sparsely monitored by the UK Department for Environment Food & Rural Affairs (Defra) measurement network. CTM also allowed us to explore the socioeconomic differentials in air pollution exposure by individual PM components as well as total PM. Another notable advantage is that our estimated mortality burdens are based on the socioeconomic and urban-rural gradient in underlying baseline mortality as well as PM_2.5_ concentration. This synthesis emphasizes the public health importance of policy measures to reduce air pollution in general in order to narrow socioeconomic differences in air pollution health burdens.

There are however also a number of disadvantages. The fact that pollutant concentrations are not derived from high density measurements but from a CTM (for a single year), means that the validated performance of the model is therefore central to the estimates we provide. A detailed spatiotemporal evaluation of the EMEP4UK model performance in comparison with measurement at background sites over the UK is reported elsewhere [54], but in brief the model represents the spatial distribution of daily measures of O_3_ and PM reasonably well at both rural and urban area (median values of Pearson’s correlation *r* across sites for rural and urban background sites 0.81 and 0.73 for O_3_ and 0.91 and 0.58 for PM_10_, respectively; 0.58 for PM_2.5_ in urban background sites only). Secondly, although the study had data with national coverage, the spatial resolution of our air pollution data (5 km grid) has limitations with regard to short-lived local pollutants such as NO_2_ and the analysis of national data for NO_2_ at this resolution does not demonstrate important aspects of socioeconomic variation. We have therefore excluded the results of NO_2_ and report only the results on pollutants such as PM and ozone whose national variation at 5 km grid resolution does, we believe, reflect important dimensions of geographical and socioeconomic variation. Although LSOAs are the areal units designed to maintain homogeneous socioeconomic demographic, their variation in areal size (smaller in urban area than 5x5km grids) suggests a 5 km gridded air pollution level might be shared by a couple of LSOAs with different socioeconomic status (mean ± SD of the SD of deprivation quintiles at residential postcodes located in a same 5 km grid: 1.5 ± 0.9 in urban area and 0.7 ± 0.6 in rural area), attenuating socioeconomic differentials in exposure to air pollution. Third, our paper also did not consider indoor air quality, the spatial variation of which may be modified by building characteristics [55], which in turn may be associated with socioeconomic position. Further misclassification of exposure may arise from assignment on the place of residence without account of the movement of people.

The fact that we used a small-area marker of socioeconomic deprivation may not be as good as the use of markers at individual or household level. However, a few studies investigated both individual and neighbourhood socioeconomic status in terms of associations with air pollution (PM_2.5,_ NO_x_ and road-traffic) and found neighbourhood socioeconomic status characteristics were more strongly associated with air pollutants than individual-level ones [15, 56, 57]. A previous study in London comparing area markers and an individual marker (from the Whitehall II cohort data) also reported that small-area markers perform well in showing variations in exposure to traffic-related air pollution (NO_x_ in this case) [39].

The concentration-mortality relationship used to quantify health burdens is the same as that used in the 2010 COMEAP report based on the US ACS study [7]. A case could be made for the use of alternative coefficients derived from a now larger world literature, including with additional studies from the UK and Europe, but the choice of coefficient is largely unimportant for assessing the *pattern* of socioeconomic variations. Additionally, we used a unique relative risk for all socioeconomic groups regardless of possible socioeconomic gradient in susceptibility (sensitivity) to air pollution-related health consequences, as there was limited evidence of differential sensitivity in England, UK. Here, we focused only on the partial susceptibility (i.e. baseline mortality rates). It would be desirable to examine the health burdens on the basis of other pollutants in addition to PM_2.5_, such as NO_2_ – especially where NO_2_ exposure levels are high. However, the basis for such calculations remains uncertain in the absence of clear quantitative evidence of the causal contribution of NO_2_ vs PM_2.5_ or other air pollutants.

Finally, it needs to be noted that the socioeconomic differentials in PM_2.5_ and associated health impacts observed in this study may not reflect the patterns in other countries of Europe or elsewhere. But the observation that substantial socioeconomic differentials may exist in mortality burdens in the absence of large variations in PM_2.5_ concentrations is likely to apply in any setting where appreciable socioeconomic differentials exist in underlying disease rates.

## Conclusions

Using nation-wide grid data of air pollution and individual mortality records linked with area-level socioeconomic deprivation, this study demonstrated modest socioeconomic differentials in fine particulate concentrations based on 5 km grid resolution data, but comparatively large differentials in associated mortality burdens because of the very strong socioeconomic gradients in underlying mortality rates. Policies or measures that reduce air pollution in general will have greater benefit in terms of the absolute burden for more deprived populations, and thus may modestly help narrow socioeconomic differences in health. Further examination coupled with simulations under different policy scenarios to reduce emissions from anthropogenic sources would help elucidate potential policy impacts.

## Additional files

Additional file 1: Maps of air pollution and socioeconomic deprivation decile groups in England. (DOCX 5346 kb)

Additional file 2: Socioeconomic gradient in PM_2.5_ exposure, baseline mortality and associated life years (LYs) lost. (DOCX 18 kb)

Additional file 3: Differentials in PM_2.5_ exposure-related life expectancy lost at birth in years (days). (DOCX 17 kb)

## Abbreviations

ACS: American Cancer Society
ASDR: age standardized death rate
CIs: confidence intervals
COMEAP: the Committee on the Medical Effects of Air Pollutants
Defra: Department for the Environment, Flood and Rural Affaires
EC: elementary carbon
EMEP: European Monitoring and Evaluation Programme
IMD: Index of multiple deprivation
LSOA: lower super output area
LYs: life years
NAEI: National Atmospheric Emission Inventory
O_3_: ozone
OM: organic matters
ONS: Office of National Statistics
PM: particulate matter
PM_10_: particulate matters with diameter < 10 μm
PM_2.5_: particulate matter with diameter < 2.5 μm
PM_2.5–10_: particulate matters with diameter in the range 2.5 to 10 μm
SS: sea salt
UK: United Kingdom
WRF: Weather Research Forecasts

## References

[1] Finkelstein MM, Jerrett M, Sears MR (2004). Traffic air pollution and mortality rate advancement periods. Am J Epidemiol.

[2] Hoek G, Brunekreef B, Goldbohm S, Fischer P, van den Brandt PA (2002). Association between mortality and indicators of traffic-related air pollution in the Netherlands: a cohort study. Lancet.

[3] Krewski D, Burnett R, Goldberg M, Hoover K, Siemiatycki J, Jerrett M, Abrahamowicz M, White WH (2000). Reanalysis of the Harvard six cities study and the American Cancer Society study of particulate air pollution and mortality.

[4] Pope CA, Burnett RT, Thun MJ, Calle EE, Krewski D, Ito K, Thurston GD (2002). Lung cancer, cardiopulmonary mortality, and long-term exposure to fine particulate air pollution. JAMA.

[5] Pope CA, Thun MJ, Namboodiri MM, Dockery DW, Evans JS, Speizer FE, Heath CW (1995). Particulate air pollution as a predictor of mortality in a prospective study of U.S. adults. Am J Respir Crit Care Med.

[6] Fischer PH, Marra M, Ameling CB, Hoek G, Beelen R, de Hoogh K, Breugelmans O, Kruize H, Janssen NA, Houthuijs D (2015). Air pollution and mortality in seven million adults: the Dutch environmental longitudinal study (DUELS). Environ Health Perspect.

[7] COMEAP (2010). The mortality effects of long-term exposure to particulate air pollution in the United Kingdom.

[8] Office for National Statistics: Trend in life expectancy at birth and at age 65 by socio-economic position based on the National Statistics Socio-economic Classification, England and Wales: 1982–1986 to 2007–2011. In Statistical Bulletin; 2015.

[9] Deguen S, Zmirou-Navier D (2010). Social inequalities resulting from health risks related to ambient air quality--a European review. Eur J Pub Health.

[10] Evans GW, Kantrowitz E (2002). Socioeconomic status and health: the potential role of environmental risk exposure. Annu Rev Public Health.

[11] O'Neill MS, McMichael AJ, Schwartz J, Wartenberg D (2007). Poverty, environment, and health: the role of environmental epidemiology and environmental epidemiologists. Epidemiology.

[12] O'Neill MS, Jerrett M, Kawachi I, Levy JI, Cohen AJ, Gouveia N, Wilkinson P, Fletcher T, Cifuentes L, Schwartz J (2003). Health, wealth, and air pollution: advancing theory and methods. Environ Health Perspect.

[13] Science for Environment Policy: Links between noise and air pollution and socioeconomic status. In: In-Depth Report 13 produced for the European Commisions, DG Environment by the Science Communication Unit, YWE. Bristol; 2016.

[14] Brainard JS, Jones AP, Bateman IJ, Lovett AA, Fallon PJ (2002). Modelling environmental equity: access to air quality in Birmingham, England. Environ Plan A.

[15] Chaix B, Gustafsson S, Jerrett M, Kristersson H, Lithman T, Boalt Å, Merlo J (2006). Children's exposure to nitrogen dioxide in Sweden: investigating environmental injustice in an egalitarian country. J Epidemiol Community Health.

[16] Havard S, Deguen S, Zmirou-Navier D, Schillinger C, Bard D (2009). Traffic-related air pollution and socioeconomic status: a spatial autocorrelation study to assess environmental equity on a small-area scale. Epidemiology.

[17] Jerrett M, Burnett RT, Kanaroglou P, Eyles J, Finkelstein N, Giovis C, Brook JR (2001). A GIS–environmental justice analysis of particulate air pollution in Hamilton, Canada. Environ Plan A.

[18] Pearce J, Kingham S (2008). Environmental inequalities in New Zealand: a national study of air pollution and environmental justice. Geoforum.

[19] Pinault L, Crouse D, Jerrett M, Brauer M, Tjepkema M (2016). Spatial associations between socioeconomic groups and NO2 air pollution exposure within three large Canadian cities. Environ Res.

[20] Hajat A, Hsia C, O’Neill MS (2015). Socioeconomic disparities and air pollution exposure: a global review. Current Environmental Health Reports.

[21] Chi GC, Hajat A, Bird CE, Cullen MR, Griffin BA, Miller KA, Shih RA, Stefanick ML, Vedal S, Whitsel EA (2016). Individual and neighborhood socioeconomic status and the association between air pollution and cardiovascular disease. Environ Health Perspect.

[22] Bell ML, Dominici F (2008). Effect modification by community characteristics on the short-term effects of ozone exposure and mortality in 98 US communities. Am J Epidemiol.

[23] Gwynn RC, Thurston GD (2001). The burden of air pollution: impacts among racial minorities. Environ Health Perspect.

[24] Jerrett M, Burnett RT, Brook J, Kanaroglou P, Giovis C, Finkelstein N, Hutchison B (2004). Do socioeconomic characteristics modify the short term association between air pollution and mortality? Evidence from a zonal time series in Hamilton, Canada. J Epidemiol Community Health.

[25] Ostro BD, Feng WY, Broadwin R, Malig BJ, Green RS, Lipsett MJ (2008). The impact of components of fine particulate matter on cardiovascular mortality in susceptible subpopulations. Occup Environ Med.

[26] Ou CQ, Hedley AJ, Chung RY, Thach TQ, Chau YK, Chan KP, Yang L, Ho SY, Wong CM, Lam TH (2008). Socioeconomic disparities in air pollution-associated mortality. Environ Res.

[27] Forastiere F, Stafoggia M, Tasco C, Picciotto S, Agabiti N, Cesaroni G, Perucci CA (2007). Socioeconomic status, particulate air pollution, and daily mortality: differential exposure or differential susceptibility. Am J Ind Med.

[28] Romieu I, Gouveia N, Cifuentes LA, de Leon AP, Junger W, Vera J, Strappa V, Hurtado-Diaz M, Miranda-Soberanis V, Rojas-Bracho L (2012). Multicity study of air pollution and mortality in Latin America (the ESCALA study). Res Rep Health Eff Inst.

[29] Hicken MT, Adar SD, Diez Roux AV, O'Neill MS, Magzamen S, Auchincloss AH, Kaufman JD (2013). Do psychosocial stress and social disadvantage modify the association between air pollution and blood pressure?The multi-ethnic study of atherosclerosis. Am J Epidemiol.

[30] Laurent O, Bard D, Filleul L, Segala C (2007). Effect of socioeconomic status on the relationship between atmospheric pollution and mortality. J Epidemiol Community Health.

[31] Zanobetti A, Schwartz J (2000). Race, gender, and social status as modifiers of the effects of PM10 on mortality. J Occup Environ Med.

[32] Zeka A, Zanobetti A, Schwartz J (2006). Individual-level modifiers of the effects of particulate matter on daily mortality. Am J Epidemiol.

[33] Huss A, Spoerri A, Egger M, Roosli M (2010). Aircraft noise, air pollution, and mortality from myocardial infarction. Epidemiology.

[34] Vieno M, Dore AJ, Stevenson DS, Doherty R, Heal MR, Reis S, Hallsworth S, Tarrason L, Wind P, Fowler D (2010). Modelling surface ozone during the 2003 heat-wave in the UK. Atmos Chem Phys.

[35] Vieno M, Heal MR, Hallsworth S, Famulari D, Doherty RM, Dore AJ, Tang YS, Braban CF, Leaver D, Sutton MA (2014). The role of long-range transport and domestic emissions in determining atmospheric secondary inorganic particle concentrations across the UK. Atmos Chem Phys.

[36] Simpson D, Benedictow A, Berge H, Bergström R, Emberson LD, Fagerli H, Flechard CR, Hayman GD, Gauss M, Jonson JE (2012). The EMEP MSC-W chemical transport model &ndash; technical description. Atmos Chem Phys.

[37] McLennan D, Barnes H, Noble M, Davies J, Garatt E (2011). The English indices of deprivation 2010.

[38] Adams J, White M (2006). Removing the health domain from the index of multiple deprivation 2004-effect on measured inequalities in census measure of health. J Public Health (Oxf).

[39] Goodman A, Wilkinson P, Stafford M, Tonne C (2011). Characterising socio-economic inequalities in exposure to air pollution: a comparison of socio-economic markers and scales of measurement. Health Place.

[40] Milojevic A, Armstrong BG, Gasparrini A, Bohnenstengel SI, Barratt B, Wilkinson P (2016). Methods to estimate acclimatization to urban Heat Island effects on heat- and cold-related mortality. Environ Health Perspect.

[41] 2011 Census, Headcounts and Household Estimates for Postcodes in England and Wales. https://www.ons.gov.uk/peoplepopulationandcommunity/populationandmigration/populationestimates/datasets/2011censusheadcountsandhouseholdestimatesforpostcodesinenglandandwales. Accessed 5 Oct 2017.

[42] Bibby P, Brindley P. Urban and Rural Classification of English Local Authority Districts and Similar Geographical Units in England: Methodology. 2016.https://www.gov.uk/government/uploads/system/uploads/attachment_data/file/539130/RUCLAD_Method__Apr_2016_.pdf. Accessed 5 Oct 2017.

[43] Royall RM (1986). Model robust confidence intervals using maximum likelihood estimators. International Statistica Review.

[44] Miller BG, Hurley JF (2003). Life table methods for quantitative impact assessments in chronic mortality. J Epidemiol Community Health.

[45] Office for National Statistics. Middle Super Output Area Mid-Year Population Estimates (supporting information). https://www.ons.gov.uk/peoplepopulationandcommunity/populationandmigration/populationestimates/datasets/middlesuperoutputareamidyearpopulationestimates. Accessed 5 Oct 2017.

[46] McLeod H, Langford IH, Jones AP, Stedman JR, Day RJ, Lorenzoni I, Bateman IJ (2000). The relationship between socio-economic indicators and air pollution in England and Wales: implications for environmental justice. Reg Environ Chang.

[47] Pearce JR, Richardson EA, Mitchell RJ, Shortt NK (2010). Environmental justice and health: the implications of the socio-spatial distribution of multiple environmental deprivation for health inequalities in the United Kingdom. Trans Inst Br Geogr.

[48] Pye S, Steadman J, Adams M, King K: Further analysis of NO2 and PM10 airpollution and social deprivation: a report produced for DEFRA. In: The National Assembly for Wales and The Northern Ireland Department of the Environment Report AEAT/ENV/R/0865. Oxfordshire: AEA Technology Environment; 2001.

[49] Wheeler BW, Ben-Shlomo Y (2005). Environmental equity, air quality, socioeconomic status, and respiratory health: a linkage analysis of routine data from the health survey for England. J Epidemiol Community Health.

[50] Briggs D, Abellan JJ, Fecht D (2008). Environmental inequity in England: small area associations between socio-economic status and environmental pollution. Soc Sci Med.

[51] Gordon M, Paul N, Karen M (2015). Who benefits from environmental policy? An environmental justice analysis of air quality change in Britain, 2001–2011. Environ Res Lett.

[52] Pye S, King K, Sturman J: Air quality and social deprivation in the UK: an environmental inequalities analysis - Final Report to Defra, Contract RMP/2035. Oxson: AEA Technology; 2006.

[53] Tonne C, Wilkinson P (2013). Long-term exposure to air pollution is associated with survival following acute coronary syndrome. Eur Heart J.

[54] Lin C, Heal MR, Vieno M, MacKenzie IA, Armstrong BG, Butland BK, Milojevic A, Chalabi Z, Atkinson RW, Stevenson DS (2016). Spatiotemporal evaluation of EMEP4UK-WRF v4.3 atmospheric chemistry transport simulations of health-related metrics for NO2, O3, PM10 and PM2.5 for 2001-2010. Geosci Model Dev Discuss.

[55] Taylor J, Shrubsole C, Davies M, Biddulph P, Das P, Hamilton I, Vardoulakis S, Mavrogianni A, Jones B, Oikonomou E (2014). The modifying effect of the building envelope on population exposure to PM2.5 from outdoor sources. Indoor Air.

[56] Cesaroni G, Badaloni C, Romano V, Donato E, Perucci CA, Forastiere F (2010). Socioeconomic position and health status of people who live near busy roads: the Rome longitudinal study (RoLS). Environ Health.

[57] Hajat A, Diez-Roux AV, Adar SD, Auchincloss AH, Lovasi GS, O'Neill MS, Sheppard L, Kaufman JD (2013). Air pollution and individual and neighborhood socioeconomic status: evidence from the multi-ethnic study of atherosclerosis (MESA). Environ Health Perspect.

