# Supplementary material for: Socioeconomic and urban-rural differentials in exposure to air pollution and mortality burden in England
Source: Environ Health. 2017 Oct 6;16:104. doi: 10.1186/s12940-017-0314-5 (PMC6389046; doi:10.1186/s12940-017-0314-5)
Supplement: Supplementary file 1 — Maps of air pollution and socioeconomic deprivation decile groups in England. (DOCX 5346 kb) [file 12940_2017_314_MOESM1_ESM.docx]

**Additional file 1. Maps of air pollution and socioeconomic deprivation decile groups in England.**

| 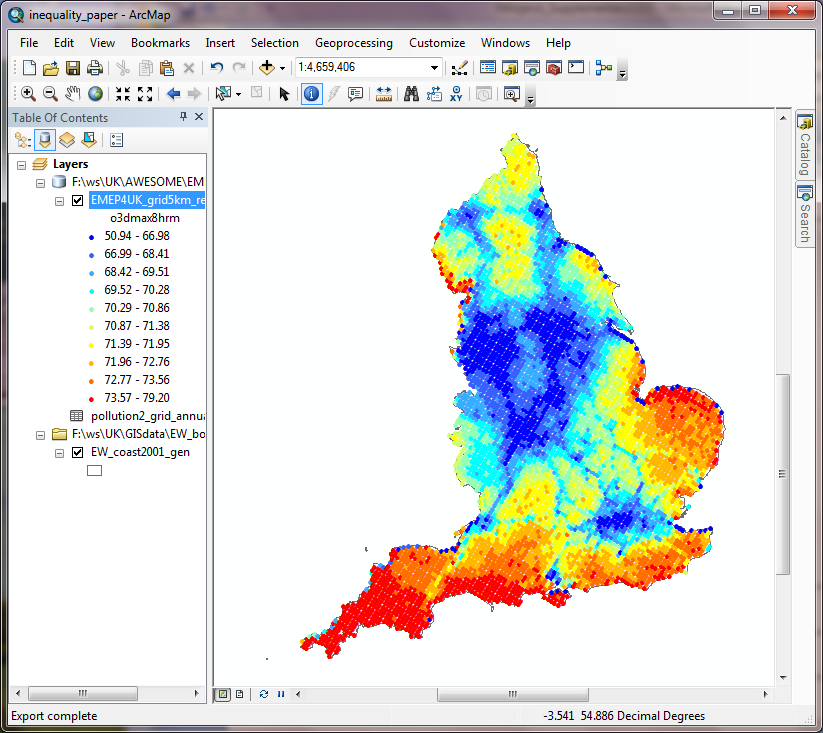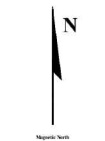 O_3_ (daily max 8hr running mean)   50 100 200 km | PM_10_  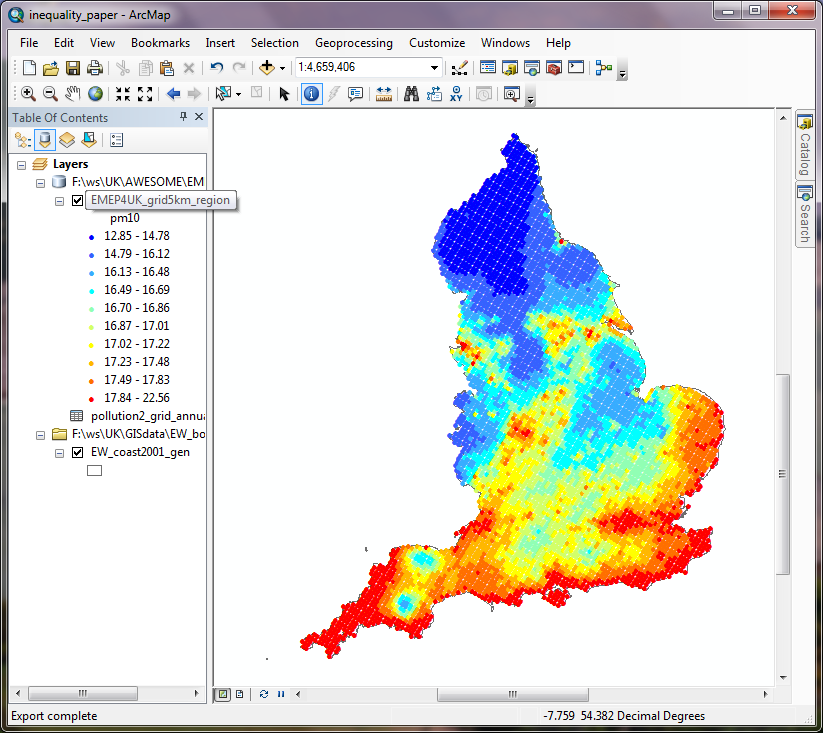 |
| --- | --- |
| PM_2.5-10_  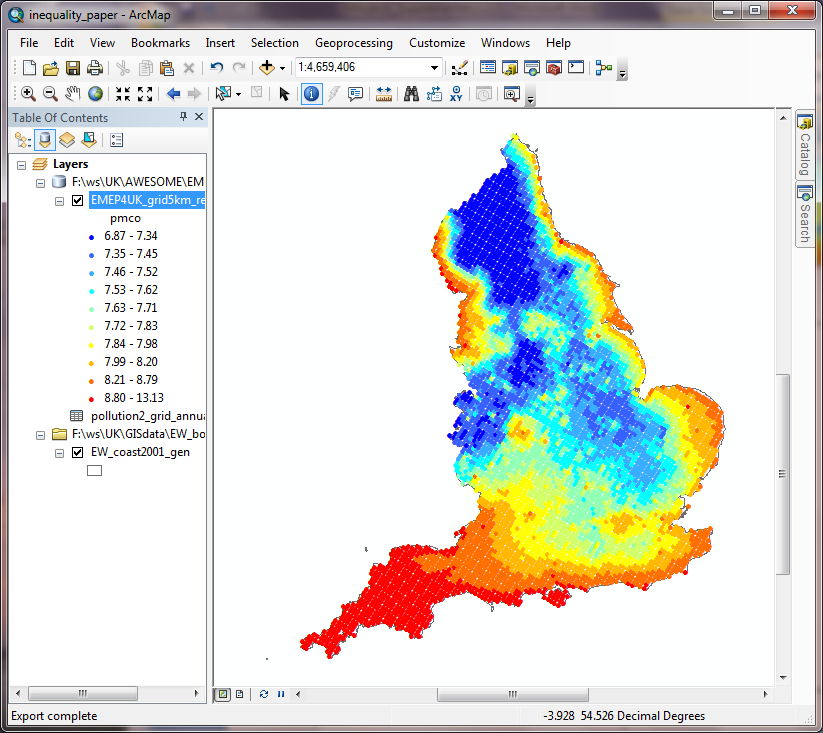 |  |

Figure 1. Annual average of daily mean O_3_, PM_10_, PM_2.5-10_, total PM_2.5_, and selected PM_2.5_ components (μg/m^3^) in England 2010, estimated by EMEP4UK model at 5km grid resolution. Graduated colours were based on decile groups of all grid values in England for each pollutant.

| PM_2.5_ (total)  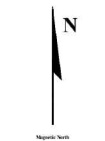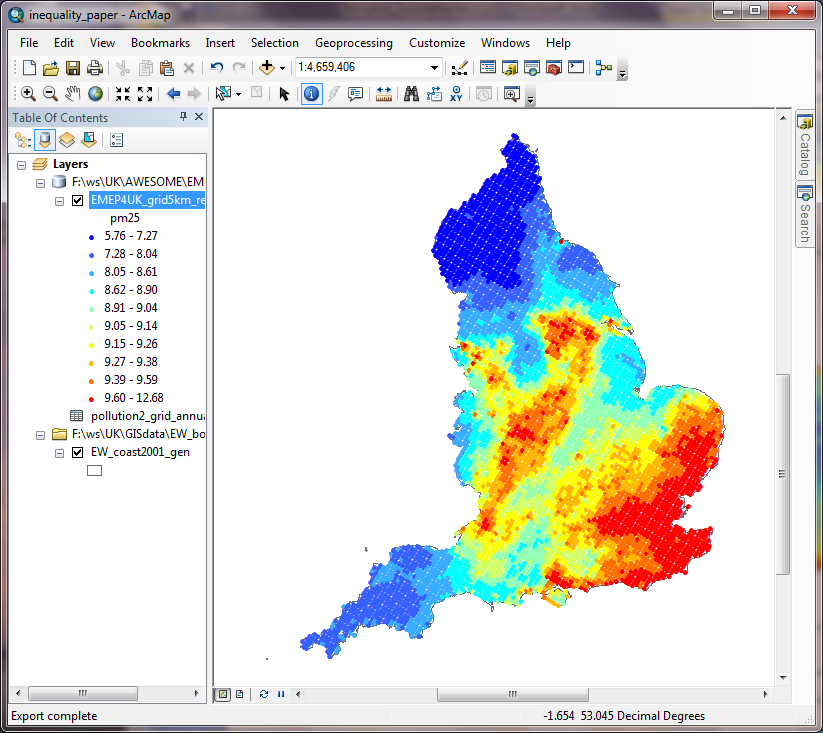  50 100 200 km | PM_2.5_ (nitrate)  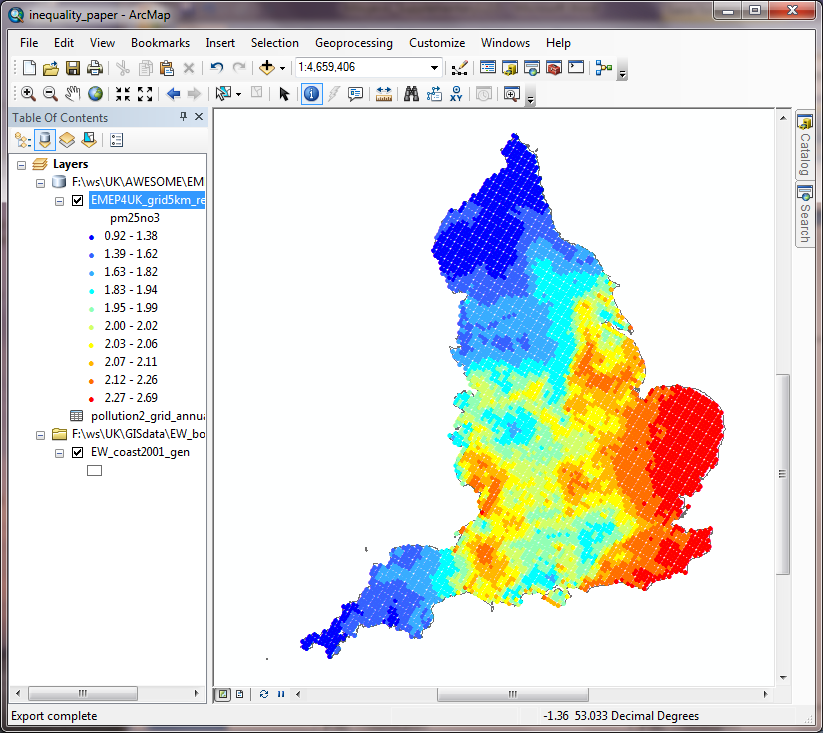 |
| --- | --- |
| PM_2.5_ (sulphate)  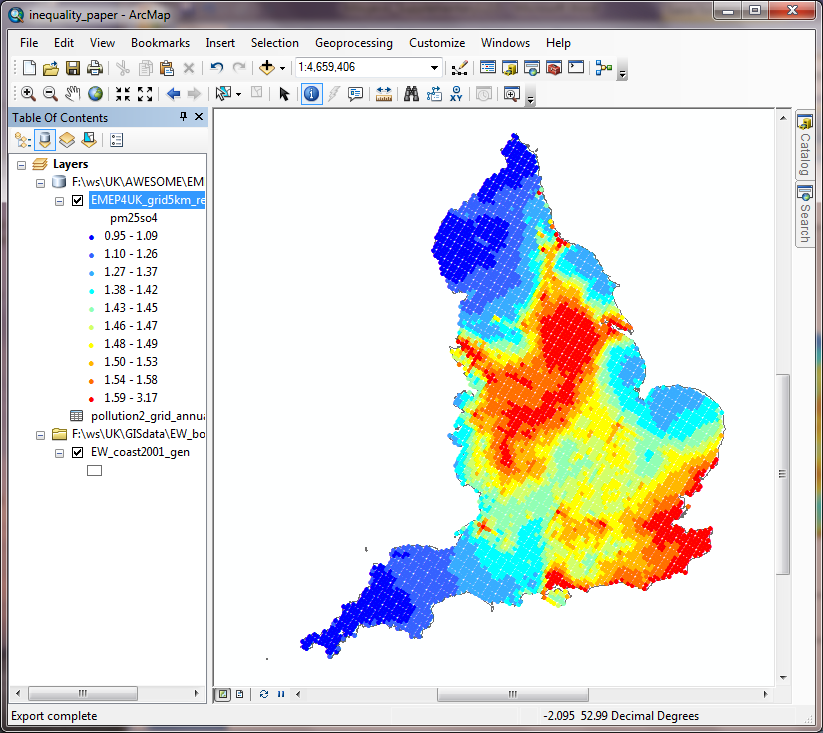 | PM_2.5_ (primary)  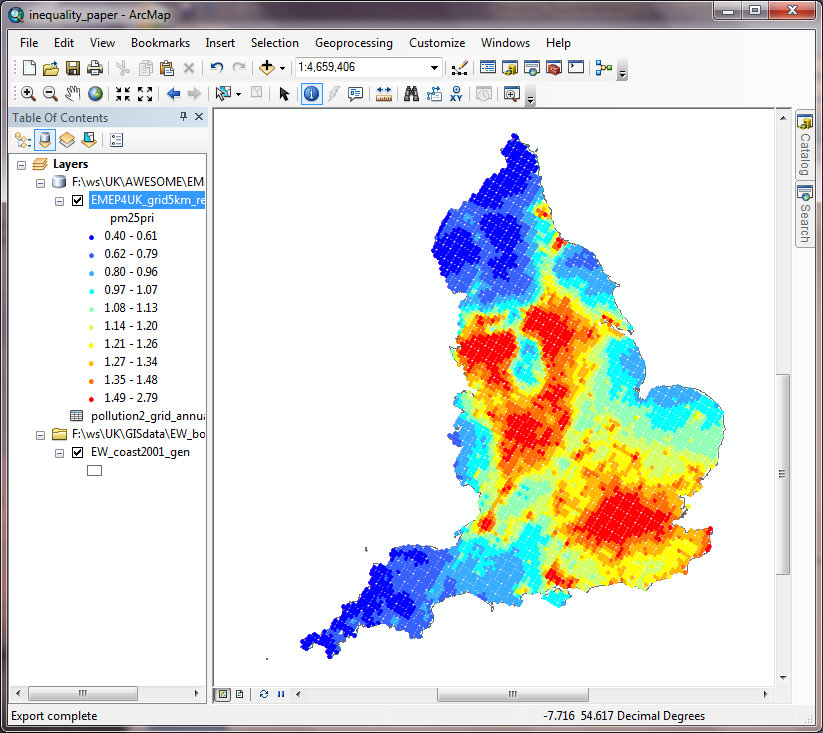 |

Figure 1. (Continued)

| 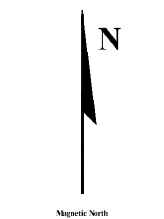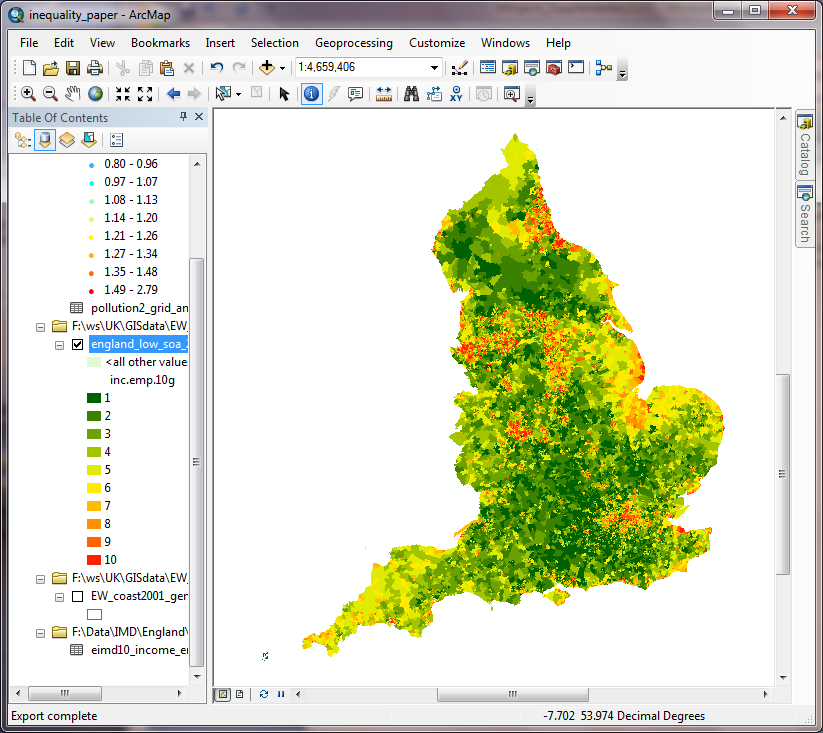  50 100 200 km  The most deprived  The least deprived |
| --- |

Figure 2. Socioeconomic deprivation decile groups in England. Socioeconomic deprivation index was reconstructed from the Income and the Employment domains in English IMD 2010 at Lower Super Output Area (LSOA) level.

Milojevic A, Niedzwieds C, Pearce J, Milner J, MacKenzie I, Doherty R, Wilkinson P: **Socioeconomic** **and urban-rural differentials in exposure to air pollution and mortality burden in England.**

50 100 200 km
