# Supplementary material for: Socioeconomic and urban-rural differentials in exposure to air pollution and mortality burden in England
Source: Environ Health. 2017 Oct 6;16:104. doi: 10.1186/s12940-017-0314-5 (PMC6389046; doi:10.1186/s12940-017-0314-5)
Supplement: Supplementary file 2 — Socioeconomic gradient in PM2.5 exposure, baseline mortality and associated life years (LYs) lost. (DOCX 18 kb) [file 12940_2017_314_MOESM2_ESM.docx]

**Additional file 2. Socio-economic gradient in PM_2.5_ exposure, baseline mortality and associated life years (LYs) lost**

| Index (unit) | Area | Deprivation deciles (1 for the least, 10 for the most) | | | | | | | | | | Ratio ^a^ |
| --- | --- | --- | --- | --- | --- | --- | --- | --- | --- | --- | --- | --- |
|  |  | 1 | 2 | 3 | 4 | 5 | 6 | 7 | 8 | 9 | 10 |  |
| Population (in 1000) ^2)^ | All | 5,118 | 5,121 | 5,104 | 5,086 | 5,093 | 5,083 | 5,089 | 5,096 | 5,100 | 5,073 | - |
|  | Urban (%) | 3,867  (76%) | 3,487  (68%) | 3,543  (69%) | 3,737  (73%) | 3,953  (78%) | 4,235  (83%) | 4,544  (89%) | 4,759  (93%) | 4,930  (97%) | 4,976  (98%) | - |
|  | Rural  (%) | 1,252  (24%) | 1,634  (32%) | 1,560  (31%) | 1,349  (27%) | 1,140  (22%) | 848  (17%) | 545  (11%) | 337  (7%) | 170  (3%) | 97  (2%) | - |
| Deaths ^b^ | All | 32,923 | 40,395 | 43,697 | 46,035 | 48,299 | 49,668 | 49,301 | 49,819 | 50,142 | 51,614 | - |
|  | Urban  (%) | 24,027  (6%) | 26,958  (7%) | 29,208  (8%) | 32,627  (9%) | 36,088  (10%) | 40,110  (11%) | 42,953  (11%) | 45,983  (12%) | 48,024  (13%) | 50,324  (13%) | - |
|  | Rural | 8,896  (10%) | 13,437  (16%) | 14,489  (17%) | 13,408  (16%) | 12,211  (14%) | 9,558  (11%) | 6,348  (7%) | 3,836  (4%) | 2,118  (2%) | 1,290  (2%) | - |
| Age-standardized death rate ^c^ (per 10^5^ population) | All | 665.8 | 805.6 | 874.1 | 916.1 | 961.1 | 979.3 | 967.9 | 964.1 | 1018.1 | 1025.3 | 1.54 |
|  | Urban | 325.9 | 407.5 | 438.5 | 465.7 | 487.1 | 502.2 | 496.8 | 501.7 | 500.9 | 516.4 | 1.59 |
|  | Rural | 339.5 | 397.7 | 434.8 | 449.5 | 473.1 | 476.0 | 470.2 | 461.3 | 515.2 | 509.9 | 1.50 |
| Annual mean of PM_2.5_ (ug/m^3^) | All | 9.175 | 9.180 | 9.186 | 9.208 | 9.202 | 9.228 | 9.272 | 9.316 | 9.366 | 9.450 | 1.03 |
|  | Urban | 9.260 | 9.273 | 9.274 | 9.307 | 9.300 | 9.314 | 9.338 | 9.365 | 9.400 | 9.470 | 1.02 |
|  | Rural | 8.925 | 8.962 | 8.973 | 8.923 | 8.872 | 8.843 | 8.862 | 8.884 | 9.025 | 9.166 | 1.03 |
| Associated LYs lost, total (yrs) | All ^d^ | 20,667 | 24,373 | 26,261 | 27,492 | 28,691 | 29,621 | 29,671 | 30,697 | 31,554 | 34,057 | 1.65 |
|  | Urban | 15,206 | 16,398 | 17,827 | 19,587 | 21,871 | 24,093 | 26,020 | 28,478 | 30,398 | 33,379 | 2.20 |
|  | Rural | 5,445 | 7,944 | 8,407 | 7,860 | 6,790 | 5,504 | 3,659 | 2,254 | 1,218 | 723 | 0.13 |
| Associated LYs lost (yrs per 1000 persons) | All ^d^ | 4.04 | 4.76 | 5.15 | 5.41 | 5.63 | 5.83 | 5.83 | 6.02 | 6.19 | 6.71 | 1.66 |
|  | Urban | 3.93 | 4.70 | 5.03 | 5.24 | 5.53 | 5.69 | 5.73 | 5.98 | 6.17 | 6.71 | 1.71 |
|  | Rural | 4.35 | 4.86 | 5.39 | 5.83 | 5.96 | 6.49 | 6.72 | 6.68 | 7.17 | 7.46 | 1.71 |

^a^ Ratio shows the most deprived group divided by the least deprived group.

^b^ Population and the number of deaths are from 2006 mid-year and total of 2006, respectively.

^c^ Adjusted for age (5 year age group) and sex by direct standardization using 2006 mid-year population in England.

^d^ Life years (LYs) lost in all area does not coincide with the figure summed up that in urban and rural area as different baseline mortality (not specific for urban or rural area) was applied for calculation.

Milojevic A, Niedzwieds C, Pearce J, Milner J, MacKenzie I, Doherty R, Wilkinson P: **Socioeconomic** **and urban-rural differentials in exposure to air pollution and mortality burden in England.**
