# Supplementary material for: Socioeconomic and urban-rural differentials in exposure to air pollution and mortality burden in England
Source: Environ Health. 2017 Oct 6;16:104. doi: 10.1186/s12940-017-0314-5 (PMC6389046; doi:10.1186/s12940-017-0314-5)
Supplement: Supplementary file 3 — Differentials in PM2.5 exposure-related life expectancy lost at birth in years (days). (DOCX 17 kb) [file 12940_2017_314_MOESM3_ESM.docx]

**Additional file 3. Differentials in PM_2.5_ exposure-related life expectancy lost at birth in years (days)**

| Deprivation deciles | All area | | Urban area | | Rural area | |
| --- | --- | --- | --- | --- | --- | --- |
|  | Male | Female | Male | Female | Male | Female |
| 1 (the least deprived) | 0.49  (179) | 0.48  (174) | 0.49  (180) | 0.48  (175) | 0.48  (175) | 0.46  (168) |
| 2 | 0.49  (179) | 0.48  (175) | 0.50  (183) | 0.49  (177) | 0.47  (172) | 0.47  (171) |
| 3 | 0.50  (182) | 0.49  (179) | 0.51  (186) | 0.50  (182) | 0.48  (176) | 0.47  (172) |
| 4 | 0.51  (187) | 0.50  (181) | 0.52  (189) | 0.50  (182) | 0.50  (182) | 0.48  (177) |
| 5 | 0.52  (188) | 0.51  (187) | 0.53  (193) | 0.52  (190) | 0.48  (174) | 0.48  (175) |
| 6 | 0.54  (196) | 0.52  (188) | 0.54  (198) | 0.52  (190) | 0.51  (186) | 0.50  (183) |
| 7 | 0.55  (200) | 0.53  (193) | 0.55  (201) | 0.53  (194) | 0.52  (190) | 0.51  (187) |
| 8 | 0.57  (207) | 0.55  (200) | 0.57  (209) | 0.55  (201) | 0.52  (190) | 0.53  (195) |
| 9 | 0.59  (217) | 0.56  (205) | 0.60  (218) | 0.56  (206) | 0.54  (199) | 0.53  (193) |
| 10 (the most deprived) | 0.62  (226) | 0.60  (220) | 0.62  (227) | 0.60  (221) | 0.53  (194) | 0.55  (199) |

Milojevic A, Niedzwieds C, Pearce J, Milner J, MacKenzie I, Doherty R, Wilkinson P: **Socioeconomic** **and urban-rural differentials in exposure to air pollution and mortality burden in England.**

50 100 200 km
